# The Mesothelial Origin of Carcinoma Associated-Fibroblasts in Peritoneal Metastasis

**DOI:** 10.3390/cancers7040872

**Published:** 2015-09-29

**Authors:** Angela Rynne-Vidal, José Antonio Jiménez-Heffernan, Concepción Fernández-Chacón, Manuel López-Cabrera, Pilar Sandoval

**Affiliations:** 1 Centro de Biología Molecular Severo Ochoa, CSIC, Universidad Autónoma de Madrid, Campus de Cantoblanco, 28049 Madrid, Spain; E-Mails: arynne@cbm.csic.es (A.R.V.); psandoval@cbm.csic.es (P.S.); 2 Departamento de Anatomía Patológica, Hospital Universitario La Princesa, Instituto de Investigación Sanitaria Princesa, 28006 Madrid, Spain; E-Mails: jjheffernan@yahoo.com (J.A.J.-H.); mcfernandezch@sanitas.es (C.F.-C.); 3 Departamento de Oncología, Hospital de la Zarzuela, 28023 Madrid, Spain

**Keywords:** mesothelial-to-mesenchymal transition, carcinoma-associated fibroblasts, peritoneal metastasis, mesothelial cells, therapeutic strategies

## Abstract

Solid tumors are complex and unstructured organs that, in addition to cancer cells, also contain other cell types. Carcinoma-associated fibroblasts (CAFs) represent an important population in the tumor microenviroment and participate in several stages of tumor progression, including cancer cell migration/invasion and metastasis. During peritoneal metastasis, cancer cells detach from the primary tumor, such as ovarian or gastrointestinal, disseminate through the peritoneal fluid and colonize the peritoneum. Tumor cells metastasize by attaching to and invading through the mesothelial cell (MC) monolayer that lines the peritoneal cavity, then colonizing the submesothelial compact zone where CAFs accumulate. CAFs may derive from different sources depending on the surrounding metastatic niche. In peritoneal metastasis, a sizeable subpopulation of CAFs originates from MCs through a mesothelial-to-mesenchymal transition (MMT), which promotes adhesion, invasion, vascularization and subsequent tumor growth. The bidirectional communication between cancer cells and MC-derived CAFs via secretion of a wide range of cytokines, growth factors and extracellular matrix components seems to be crucial for the establishment and progression of the metastasis in the peritoneum. This manuscript provides a comprehensive review of novel advances in understanding how peritoneal CAFs provide cancer cells with a supportive microenvironment, as well as the development of future therapeutic approaches by interfering with the MMT in the peritoneum.

## 1. Introduction

The majority of tumors are confined to the organ where they first originated and are usually treatable and curable with local therapy. However, several neoplasias are incurable due to processes that govern the ability of cells to disseminate and implant in distant locations. Although hematogenous and lymphatic dissemination are the most common routes for metastasis, tumors originating adjacent to the peritoneal cavity, such as ovarian or colorectal cancer, frequently disseminate via transcoelomic route to develop peritoneal metastases. Cancer cells detached from the primary tumor are transported by peritoneal fluid to subsequently spread locally colonizing the peritoneum [1,2]. This process is known as peritoneal carcinomatosis and signifies that the disease is at advanced stage, is difficult to treat and, often, there is no prospect of cure. A common characteristic of abdominal cancers that progress with peritoneal metastasis is that they generally evolve very rapidly and correlate with poor prognosis. The peritoneal cavity is the site of metastases in up to 28% of recurrent endometrial cancers [3], 42% of colorectal cancers [4], 40% of gastric cancers [5] and 70% of ovarian advanced cancers [2]. Surgery is inefficient to render patients free of disease, resulting in low survival rates. This is in part due to the diffuse nature of peritoneal metastases, which renders them generally intractable to surgical resection. Nowadays, aggressive surgical tumor removal (tumor cytoreduction) coupled with hyperthermic intraperitoneal chemotherapy (HIPEC) represents the cornerstone of advanced abdominal oncologic surgery. The combination of both treatments seems to be encouraging and in colorectal and gynecological cancers provides five-year survival rates of over 40% and 45%, respectively [6,7]. However, these are complex therapies that require specialized technological facilities with highly qualified human resources and the possibility of curing advanced stage intraperitoneal tumors still remains limited [8]. Part of this problem derives from the fact that the pathogenesis of peritoneal carcinomatosis is not well understood. Therefore, to design new therapeutic approaches it is necessary to improve our knowledge of the mechanisms implicated in tumor progression in the peritoneum. This manuscript focuses on providing a comprehensive review of novel advances in understanding how the peritoneal stroma and, in particular, carcinoma-associated fibroblasts (CAFs) provide cancer cells with a supportive microenvironment for peritoneal implantation. In this context, the recent description of the mesothelial origin of peritoneal CAFs via mesothelial-to-mesenchymal transition (MMT) opens a new research line to be considered in the treatment of peritoneal metastases that frequently occur in patients with abdominal cancers [9].

## 2. The Seed and Soil Theory in Peritoneal Metastasis

Metastasis remains the major cause of death for cancer patients. Cancers that metastasize are generally more difficult to treat and have a worse prognosis than those that remain at the site of origin [10]. During peritoneal metastasis, cancer cells leave the primary tumor to colonize the secondary organ (peritoneum) where they develop into metastatic lesions. The peritoneum is a well-known metastatic site for several intra-abdominal malignancies, such as ovarian, colon, gastric, pancreatic, endometrial and rectal cancer. However, the specific site of distant metastasis is not simply due to the intrinsic features of the primary tumor, its anatomic location or proximity to secondary sites but, rather, it involves interactions between tumor cells and the local microenvironment at the secondary site. Therefore, in the establishment of metastasis, the properties of the tumor are as important as the metastatic niche. In 1899, Stephen Paget suggested the “seed and soil” hypothesis to explain how sites where metastases occur are defined not only by the tumor cell (“seed”) but also the microenvironment of the secondary metastatic site (“soil”). Accordingly, metastases are influenced by innumerable factors and complex cellular interactions between the seed and the soil [11,12]. In the case of peritoneal carcinomatosis, the metastatic niche is composed of the surface of the peritoneum overlying the abdominal cavity. The organization of the peritoneum is simple: a single layer of mesothelial cells (MCs) lines a compact region that is composed of connective tissue with a few fibroblasts, mast cells, macrophages and vessels [13]. The “seed and soil” mechanism seems to be especially relevant in peritoneal metastasis, since profound modifications of the surrounding metastatic stroma have been recently described during the processes of attaching to and invading through the peritoneal membrane.

The transformed peritoneal microenvironment forms a suitable niche for peritoneal metastasis, promoting the progression of secondary tumors [9]. Therefore, it is tempting to speculate that future therapeutic options for the treatment of peritoneal metastasis may lie in modulating the interaction between the tumor and the mesothelium by regulating the molecules that modify the metastatic microenvironment.

## 3. Carcinoma-Associated Fibroblasts in the Peritoneum

Currently, there is an increased understanding of the processes that govern the malignant transformation of cells. It is known that tumor development is mainly associated with the accumulation of multiple genetic and epigenetic alterations in cancer cells which pave the way for the transformation of a normal cell into a malignant cell [14]. However, tumors are highly complex organs composed of different cell types. Cancer tissue consists of both tumor cells and stromal cells surrounded by an extensive extracellular matrix (ECM). The stromal component constitutes a large part of most solid tumors and includes multiple cell types, such as mesenchymal, vascular and immune cells, that converge to support a tumorigenic niche. The development of the tumor microenvironment is triggered by signaling molecules secreted by cancer cells that corrupt the adjacent normal tissue to create an “activated” stroma [15].

The peritoneal pre-metastatic niche is basically composed of MCs, fibroblasts, endothelial cells, adipocytes and immune cells, including macrophages. However, during peritoneal metastasis normal mesothelium is replaced by a strong stromal reaction (desmoplasia) characterized by the accumulation of activated fibroblasts (myofibroblasts) [16]. The exacerbated accumulation of activated fibroblasts in the peritoneum has been previously described in other fibrotic disorders including peritoneal dialysis-induced fibrosis [17] and abdominal adhesions [18]. In the cancer stroma, it has become clear that activated fibroblasts are prominent modifiers of tumor progression towards advanced stages and their presence is often associated with poor clinical prognosis for oncology patients. They are known as carcinoma-associated fibroblasts (CAFs)—among other terms, such as tumor-associated fibroblasts or reactive stromal fibroblasts—and represent a predominant population in the tumor architecture [19]. CAFs share characteristics with myofibroblasts, such as the expression of alpha-smooth muscle actin (α-SMA) [20,21,22], fibroblast activation protein-alpha (FAP-α) [23,24] and fibroblast-specific protein 1 (FSP1) [25,26]. They are activated fibroblasts capable of producing a wide array of cytokines, growth factors, proteases, and ECM components, thereby contributing to increasing the motility and invasive potential of tumor cells. Additionally, CAFs have been widely implicated in promoting tumor angiogenesis, which is necessary for cancer survival and metastasis towards secondary organs [27,28].

## 4. Mesothelial Cells as Source of Carcinoma-Associated Fibroblasts

An important effect of the tumor nesting into the peritoneal membrane is the exacerbated accumulation of CAFs [16]. However, the multiple origins of peritoneal CAFs are still debated today. It has been proposed that CAFs may derive from different sources depending on the surrounding tumor metastatic niche. Additionally, there is emerging evidence that the origin of CAFs may vary between cancer types and within different areas of individual tumors [29]. The activation of resident fibroblasts was considered the main origin of CAFs in the tumor microenvironment [30]. However, recent studies have revealed that bone marrow-derived stem cells (fibrocytes) are integrated into tumor stroma and differentiate into myofibroblasts. Cancer cells can also undergo an epithelial-to-mesenchymal transition to acquire myofibroblast properties, although their contribution to the CAF population is small [29,31,32]. In addition, it has been shown that endothelial cells, through an endothelial-to-mesenchymal transition, may also be a source of CAFs [33,34]. Moreover, MCs have been traditionally considered as an important source of activated fibroblasts in pathologies that present fibrosis. The presence of MCs converted into myofibroblasts though MMT was first described in the peritoneum of peritoneal dialysis patients [35]. Emerging evidence has subsequently revealed that MMT is an important event in numerous fibrogenic disorders such as idiopathic lung fibrosis [36], liver fibrogenesis [37] and myocardial infarction scars [38,39]. However, until recently, it was unclear whether normal MCs convert into CAFs during peritoneal carcinomatosis. The presence of a sizeable subpopulation of CAFs originated from MCs through MMT in patients with malignant peritoneal disease was first reported in 2013 [9]. Evidence of MMT was based on the immunohistochemical detection of specific mesothelial markers (calretinin, cytokeratins, WT1, mesothelin) in submesothelial myofibroblasts (α-SMA) within or near peritoneal implants from ovarian and colon cancers. Supporting these data, FAP-positive mesothelium in gastric cancer patients has been associated with advanced stage disease, peritoneal dissemination and poor survival [40].

Briefly, during MMT the MCs lose their epithelial characteristics to acquire a myofibroblastic phenotype [35,41]. MMT is a complex and stepwise process that involves alterations in the cellular architecture and a deep molecular reprogramming. The MMT starts with the dissociation of intercellular junctions, due to downregulation of intercellular adhesion molecules, and with the loss of microvilli and apico-basal polarity. Then, cells adopt a front-to-back polarity, acquire α-SMA expression and increase their migratory capacity. In the last stages of the MMT, the cells acquire the capacity to degrade the basement membrane and to invade the fibrotic compact zone [42]. During the end stages of myofibroblast conversion, MCs are able to produce a large amount of extracellular matrix components and synthesize a wide range of inflammatory, profibrotic and angiogenic factors that may contribute to structural and functional changes in the peritoneal membrane [35,41,43,44,45,46].

## 5. Mesothelial-to-Mesenchymal Transition Promoting Stimuli in Peritoneal Metastasis

Peritoneal metastasis is a consequence of a sequential process that can be briefly summarized in three principal steps: (a) Tumor cells trigger an early MMT and adhere to mesothelial monolayer; (b) MMT progresses and tumor cells invade through the peritoneum; (c) Mesothelial-derived CAFs accumulate in the submesothelial compact zone, where they provide the tumor with the adequate blood support and ECM components to progress. Therefore, the myofibroblastic conversion of MCs plays an important role in both the initial stages of peritoneal metastasis and the growth of the secondary tumor implants [9].

In the context of peritoneal metastasis, the promoting stimuli able to induce the mesenchymal conversion of MCs are still not completely characterized. An important characteristic of patients that develop peritoneal carcinomatosis is the presence of ascites, the pathologic accumulation of fluid in the peritoneal cavity. In this context, ovarian cancer can account for up to 47% of malignant ascites, followed by other gastrointestinal cancers; all of them correlating with poor survival, gastrointestinal tumors being the most severe [47,48,49]. Malignant ascitic fluid is composed of cytokines, chemokines, growth factors, exosomes and suspended cells that vary in proportion between patients: leukocytes, MCs, macrophages, tumor cells and plasma cells [50,51,52,53,54]. Many cytokines and growth factors are present in high concentration in malignant ascitic fluid, including transforming growth factor-beta1 (TGF-β1), tumor necrosis factor-alpha (TNF-α), vascular endothelial growth factor (VEGF), hepatocyte growth factor (HGF), interleukin-1 beta (IL-1β), interleukin-6 (IL-6), interleukin-8 (IL-8) and interleukin-10 (IL-10) [55,56,57,58,59,60,61,62]. Interestingly, many of these soluble mediators, such as TNF-α, IL-6, TGF-β1 and IL-1β have important pro-inflammatory roles in the peritoneal environment and, as a consequence, have been widely implicated in fibrosis by stimulating fibroblast proliferation [63,64], ECM component deposition, and MMT induction [41,65]. Therefore, it is tempting to speculate that in peritoneal metastasis diverse cytokines could be contributing to the modification of the mesothelial surface in order to initialize tumor implantation [66,67,68]. To this effect, elevated IL-6 and TNF-α in ascites of patients in late stages of ovarian cancer are considered independent predictors of poor survival [67,69].

Among the growth factors accumulated in ascites, HGF has recently been implicated in the implantation of endometrial cells [70] and ovarian cancer cells [71] in the peritoneum via a MMT. Regarding this idea, *in vitro* experiments have shown that ovarian cancer ascites enhances the migration and invasion of both patient-derived peritoneal MCs [55] and ovarian cancer cells [56] through HGF-dependent mechanisms.

TGF-β1 is a master molecule that accumulates in the ascitic fluid and presents both anti- and pro-tumoral effects [72,73]. However, TGF-β1 is also a prototypical inducer of MMT and is a key factor in the activation of peritoneal fibroblasts, regardless of their origin [74]. The activity of TGF-β1 on stromal cells has been reported to increase the efficiency of organ colonization by tumor cells [23]. Thus, it can be speculated that targeting the TGF-β1 pathway could interfere with the accumulation of peritoneal CAFs [75]. A large proportion of tumors, including colorectal and ovarian cancer, display mutational inactivation of the TGF-β1 pathway yet, paradoxically, they are characterized by elevated TGF-β production [23]. In fact, *in vitro* experiments have showed that carcinoma cells secrete high concentrations of TGF-β1, inducing the mesenchymal conversion of MCs. In addition, blockade of the TGFβ type I receptor prevents the conversion of MCs into CAFs mediated by tumor conditioned media [9]. Similarly, Miao *et al.* demonstrated that gastric cancer cells expressing high levels of TGF-β1 induce both downregulation of E-cadherin and upregulation of α-SMA in the mesothelium [76].

One general characteristic of tumors is their ability to release vesicular portions of membrane material, termed exosomes, which were initially described by Thery *et al.* [77]. Exosomes serve as vehicles that transfer proteins, as well as RNA (mRNA and miRNA), between cells and have been found in malignant ascites from ovarian and gastrointestinal cancer patients [52,78,79,80]. While the precise mechanism of communication between cancer and MCs in the peritoneum remains unclear, there is growing evidence that exosomes may serve as prognostic/diagnostic indicators of peritoneal dissemination. Regarding this idea, exosomes derived from colorectal cancer ascites contain proteins that may promote tumor progression via angiogenesis, disruption of epithelial cell polarity, immune modulation, tumor growth and invasion [52]. On this note, ovarian cancer exosomes administered to mice prior to tumor cell injection have been shown to induce a more aggressive disease and to increase tumor growth [81]. Of the miRNAs contained in exosomes, miR-21, known for its pro-oncogenic activity [82], is present in both ovarian [79] and gastric [80] cancer ascites. Expression of miR-21 in exosomes is associated with pathways related to TGFβ signaling, ECM-receptor interaction, mesothelial clearance and worse prognosis/diagnostic value; thus, providing a novel approach for early diagnosis of peritoneal dissemination [80,81]. In addition, Vaksman *et al.* concluded that the effect of exosomes is mainly exerted on MCs, rather than on tumor cells, and higher miRNA levels are associated with poor survival [81]. These data suggest that exosomes may play a role in modifying the metastatic niche to favor peritoneal dissemination.

## 6. Implication of Mesothelial-Derived Carcinoma-Associated Fibroblasts in Adhesion, Invasion and Progression of Peritoneal Metastasis

Independently of the MMT-promoting factors that could initiate peritoneal metastasis, different hypotheses have tried to explain how malignant cells attach to the peritoneal membrane during the earliest stages. Initially, it was believed that MCs were simply victims of tumor aggression to the peritoneum [83,84]. Some experimental models proposed that intraperitoneal cancer spheroids gain access to the submesothelium by exerting force to clear MCs [85,86,87]. Subsequently, there was speculation that cancer cells do not adhere to the MCs but, rather, to the connective tissue under the MCs. In this context, “milky spots” are omental areas in which the ECM is exposed and they have been identified as the preferred sites for peritoneal colonization [88]. However, “milky spots” are structures essentially comprised of an accumulation of immune cells and capillaries where the triggering of an inflammatory response can influence peritoneal metastasis, transforming the initial pattern of the “milky spot” attachment into a widespread pattern of dissemination [89]. On the other hand, *in vitro* adhesion experiments have demonstrated that cancer cells bind better to mesenchymal than to epithelial-like MC monolayers [9,40]. Scanning electron microscopy analysis revealed that the enhanced adhesion of tumor cells to mesenchymal MCs is not due to a mere exposure of the underlying matrix but rather to an increased cell-cell interaction [9]. De Vlieghere *et al.* have recently shown that CAFs are able to selectively capture floating cancer cells, delaying tumor adhesion to the natural mesothelial niche [90]. Then, MMT may play an important role in conferring an advantage on metastatic cells for attachment to the peritoneal membrane [9]. A wide range of adhesion molecules including CD44, CA125/MUC16, ICAM-1 or VCAM-1 are reportedly involved in the binding of cancer cells to the mesothelium [91,92,93,94,95,96,97]. The molecular mechanisms that mediate the tumor-mesothelium interaction during the mesenchymal conversion of MCs would require further analysis. However, a growing body of experimental evidence on adhesion concurs that, at initial stages of peritoneal metastasis, MMT enhances the binding of cancer cells on the peritoneum in a β1-integrin-dependent manner [9,98,99]. In agreement with this notion, it has been reported that cleavage of MC-associated matrix proteins fibronectin and vitronectin by MMP-2 enhances integrin-mediated carcinoma-mesothelium attachment [100,101]. Furthermore, blocking antibodies or siRNA directed against either ICAM-1 [102], VCAM-1 [103], fibronectin [101] or their integrin ligands significantly decrease the adhesion and invasion of ovarian cancer cells through the MC monolayer, and dramatically reduced metastases in a mouse model [104].

In a second stage of peritoneal metastasis, tumor cells reach the submesothelial compact zone. Histological analysis has shown that MC-derived CAFs accumulate in areas with micrometastases but not in tumor-free regions, suggesting that tumor cells promote the invasion of adjacent MCs. Indeed, *in vitro* invasion assays suggest that carcinoma cells embedded in a matrix enhance the invasive capacity of MCs. The enhanced invasion triggered by tumor cells seems to be a consequence of the acquisition of a mesenchymal phenotype by MCs [9]. In fact, during the MMT process, MCs increase their migration/invasion capacity [105]. Therefore, MCs that have invaded the matrix, in turn, further promote invasion by carcinoma cells. Mesenchymal MCs and carcinoma cells seem to establish a feed-forward cycle by mutually stimulating their invasive capacity. Accordingly, MCs have been considered as the advancing front of the tumor in the processes of peritoneal carcinomatosis [106,107]. MCs behave in a similar fashion to resident fibroblasts in terms of the induction of carcinoma cell invasion *in vitro*. It has been shown that normal omentum fibroblasts induce the adhesion and invasion of carcinoma cells in 3D culture models [101,108]. On the other hand, Cai *et al.* reported that activated fibroblasts (either CAFs isolated from omentum of patients with ovarian cancer metastasis or normal omentum fibroblasts stimulated with TGF-β1) have stronger effects on carcinoma cell attachment and invasion than normal fibroblasts [109]. Furthermore, the growth dynamics of cancer xenografts produced in response to intraperitoneal co-injection of omental MCs with either ovarian [110] or gastric [111] cancer cells was remarkably greater than for implantation of cancer cells alone.

Therefore, in stark contrast to initial impressions, it is now accepted that MCs are not a passive barrier that prevents the progression of the tumor into the peritoneum, but are able to actively promote the process of adhesion to and invasion through the peritoneum.

In order for tumor cells to migrate and/or invade through the colonized organs, ECM disruption is crucial [112]. Matrix metalloproteinases (MMP) are known to be important molecular players in the physiological processes of cancer progression by facilitating angiogenesis and tumor invasion through matrix degradation [113,114,115]. In this context, CAFs have been widely implicated in the remodeling of the ECM components through MMPs to consequently favor cancer invasion [116,117]. It is well known that, at an intermediate stage of epithelial-to-mesenchymal transition, cells acquire the capacity to degrade the basement membrane and to invade the fibrotic stroma by upregulating the expression of MMPs [42]. In MCs, the expression of MMP-2 and MMP-9 increases in response to MMT-inducing stimuli such as TGF-β [118] or TNF-α [119]. Interestingly, both of these MMPs can activate the latent form of TGF-β [120]. In addition, fibroblasts in omentum that have been activated by tumor cells have been shown to promote ovarian invasiveness via MMP-2 overexpression [109]. Similarly, exosomes of ovarian cancer ascites contain MMP-2, MMP-9 and urokinase-type plasminogen activator (uPa), all of them able to degrade the ECM, facilitating invasion and dissemination of tumor cells [53]. Additionally, expression of VEGF by ovarian cancer cells is involved in transforming the ECM by stimulating MMP-9 expression at the tumor site [121]. With regard to the peritoneal environment, MCs undergoing a MMT contribute to the deposition of ECM components, including fibronectin and collagen I [9]. Therefore, although further analysis is required, we could speculate that, by increasing ECM deposition and MMP expression, mesothelial-derived CAFs regulate their own invasive capacity while helping to provide the appropriate ECM scaffold for cancer cells to survive in the metastatic niche [122,123,124,125].

CAFs in the tumor compartment have been implicated in promoting the growth and/or proliferation of cancer cells, ensuring their survival at the colonized organ. Although, mesothelial-derived CAFs have not yet been implicated in directly augmenting tumor growth, some authors have suggested that MCs establish communication with the tumor by secreting proliferating stimuli or by direct cell–cell contact which, in turn, stimulate proliferation of cancer cells and drive peritoneal dissemination [110,111].

**Figure 1:**
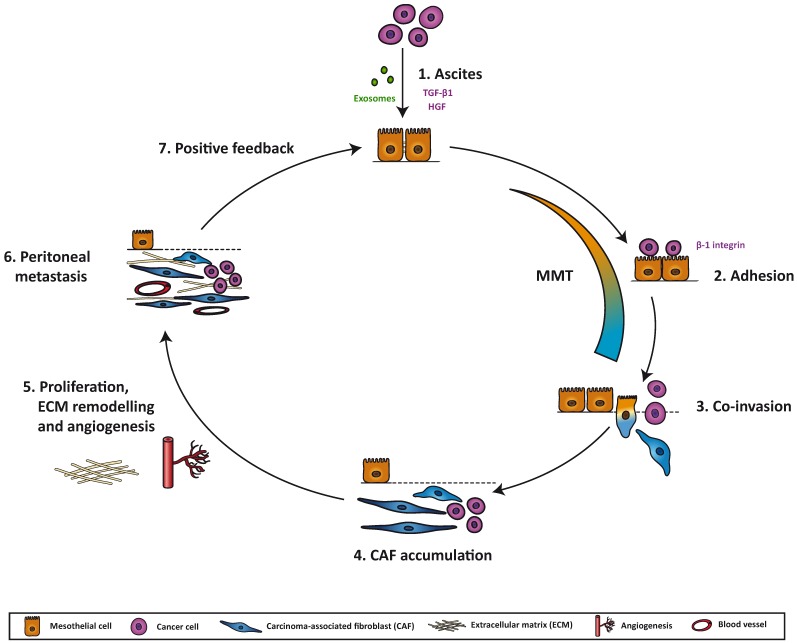
Model for transformation of the pre-metastatic niche by mesothelial-to-mesenchymal transition in the peritoneum: (1) Cancer cells at the primary site secrete an array of exosomes, cytokines, chemokines and growth factors that accumulate in the ascitic fluid. Some of these molecules, such as transforming growth factor-beta1 (TGF-β1) or hepatocyte growth factor (HGF), induce changes in the mesothelial cells (MCs), triggering a mesothelial-to-mesenchymal transition (MMT), through which they lose apico-basal polarity and cell-cell adhesion, and acquire myofibroblastic properties. (2) Cancer cells adhere via β1 integrins to the MCs that line the peritoneum. This adhesion is increased when a MMT has taken place. (3) In later stages of MMT, MCs have converted into carcinoma-associated fibroblasts (CAFs) that represent the invasion front into the stroma, followed by cancer cells. (4) After invading, MC-derived CAFs accumulate in the peritoneal stroma. (5) CAFs produce factors that affect peritoneal implant progression: they induce angiogenesis via secretion of VEGF, among other factors; they transform the extracellular matrix (ECM) by producing collagen, fibronectin and other structural proteins, and they remodel the ECM via matrix metalloproteinases (MMPs); they also stimulate proliferation of cancer cells. (6) The metastatic niche in the peritoneum is created as a result of the clearance of the MC monolayer, myofibroblast conversion of MCs, invasion of MCs and cancer cells, accumulation of CAFs, increased vascularization, ECM remodeling and proliferation of cancer cells. (7) Cancer cells in the new site, together with CAFs, will continue to produce factors that modify the stroma and induce changes in both cancer cells and MCs, thereby creating a positive feedback loop.

At advanced stages of peritoneal metastasis, vascularization is necessary for tumor progression. Angiogenesis is also influenced by the organ microenvironment and is the result of an imbalance between positive and negative angiogenic factors released by tumor and host cells into the microenvironment of the neoplastic tissue [126]. In ovarian cancer progression in particular, the key role of vascular endothelial growth factor (VEGF) has been well established and is correlated with decreased overall survival [127]. Interestingly, VEGF appears as one of the most induced factors in MCs transformed into myofibroblasts [128,129]. The fact that the MCs that have undergone MMT express high amounts of VEGF suggests that peritoneal CAFs may play an important role in tumor vascularization [130]. In this respect, prominent new vessel formation has been observed in the peritoneal areas of human biopsies harboring tumor cells and MC-derived CAFs when compared to tumor-free regions. Indeed, the use of a mouse model of peritoneal dissemination confirmed increased angiogenesis at tumor implant sites [9]. In fact, Sako *et al.* showed that peritoneal dissemination of gastric cancer was significantly suppressed when adenovirus expressing a soluble form of the VEGF receptor Flt-1 was administered intraperitoneally to mice [131].

In summary, peritoneal metastasis is the result of a close multi-directional communication between cancer cells, MCs and CAFs. Tumor cells are able to trigger the myofibroblastic conversion of MCs via MMT, thus increasing adhesion to and invasion through the peritoneum. Finally, mesothelial-derived CAFs accumulated in the submesothelial compact zone provide the tumor with the adequate blood support, ECM components and/or proliferating signals to progress to advanced stages (Figure 1).

## 7. Mesothelial-to-Mesenchymal Transition as a Potential Therapeutic Target in Peritoneal Metastasis

CAFs’ proven tumor-promoting capacities have raised interest in exploiting them as drug targets for anticancer therapy [132]. Undoubtedly, the recent description of the origin of CAFs from the adjacent mesothelium via MMT could point to an alternative target in the treatment of metastases that disseminate via the peritoneum, since this process can be easily modulated. Therapeutic strategies could be designed to avoid the accumulation of mesothelial-derived CAFs in the compact zone by preventing or reverting the MMT itself, by reducing the MMT-promoting stimuli in the peritoneal cavity, and/or by treating the MMT-associated effects, such as tumor cell adhesion and invasion, ECM accumulation or pro-angiogenic factor synthesis.

MMT results from molecular signals accumulated in the peritoneal cavity. These include inflammatory cytokines and growth factors [133]. Therapeutic approaches could be directed to interfere with or modify the upstream MMT-promoting stimuli operating in the peritoneal cavity prior to tumor adhesion to the mesothelium. Regarding this idea, TGF-β1 is a master molecule in the induction of MMT and is highly accumulated in malignant ascitic fluids. It has been demonstrated that interfering with the TGF-β1 pathway *in vitro* prevents the conversion of MCs into CAFs [9]. *In vivo* experiments also showed that using a TGF-β1 blocking agent prevented peritoneal dissemination of gastric cancer cells by preserving the mesothelial monolayer structure [76]. Although further studies are needed in this regard, it should be considered that agents directly blocking TGF-β1 cannot be easily employed in the clinical practice, at least for long-term treatments, because TGF-β1 has important modulating functions of the immune and inflammatory responses [133,134]. Hence, the molecular studies of the downstream TGF-β1 Smad-dependent signaling pathways involved in MMT could provide more specific strategies for the preservation of peritoneal membrane with fewer side effects. In this context, the use of an endogenous negative regulator of this process, bone morphogenetic protein (BMP)-7, has been shown to block/reverse MMT *in vitro*, *ex vivo* and *in vivo*, reducing fibrosis, angiogenesis, invasion and the acquisition of a mesenchymal phenotype by MCs [135,136]. The molecular characterization of the TGF-β1-mediated Smad-independent signaling involved in the prevention/reversion of MMT could provide a wide range of possible molecular targets with roles in regulating the MMT, such as transforming growth factor-activated kinase 1 (TAK1) [105], extracellular-signal-regulated kinases (ERKs)-1/2 and the nuclear factor-κB (NF-κB) [137,138,139].

As discussed above, MMT could result from molecular inflammatory signals associated with the intraperitoneal ascitic environment. Previous studies of MMT in peritoneal dialysis have demonstrated that the use of pharmacological agents that target inflammation preserve the peritoneal membrane. Celecoxib is a potent anti-inflammatory drug whose mechanism of action is based on the inhibition of cyclooxygenase (COX)-2, which is expressed at high levels by MCs that undergo MMT. COX-2 has been widely implicated in inflammatory responses, as well as in fibrotic and angiogenic processes in animal peritoneal dialysis models [140,141]. Interestingly, expression of COX-2 is also elevated in tumor cells from colorectal and ovarian carcinomas [142,143]. Multiple clinical studies show the effectiveness of specific inhibitors of COX-2 in preventing or delaying re-occurrence of cancer, including metastases, in high risk patients [144,145,146,147].

Targeting adhesion of cancer cells to the mesothelium could also be considered as a therapeutic strategy for treatment of peritoneal metastasis, given that the use of blocking antibodies or siRNA against α5 and/or β1 integrins has shown significantly reduced attachment, tumor burden and peritoneal metastasis in mouse models [104,148,149,150,151]. Interestingly, it has been reported that the oxidative stress that accompanies senescent MCs may facilitate the adhesion and dissemination of cancer cells; and the use of an antioxidant significantly reduced this attachment [95,152]. Similarly to the MMT, senescence mediates the interaction of tumor cells and MCs via binding of α5β1 integrin to fibronectin, respectively [152]. Additionally, higher production levels of TGF-β1 by MCs have been associated with oxidative stress, but also with age of the patients, suggesting there may be an accumulation of senescent MCs *in vivo* [153].

An alternative approach could be the use of pharmacological agents that preserve the mesothelium. With regard to ovarian cancer, hormone treatment with Tamoxifen is often used in conjunction with chemotherapy and other therapies [154]. Tamoxifen is a synthetic modulator of the estrogen receptor that is known to act in peritoneal tissue. Its efficacy in preserving the peritoneal structure was tested in a mouse model of peritoneal dialysis, where it reduced peritoneal thickness, angiogenesis and invasion of the compact zone by transdifferentiated MCs. The mechanism by which Tamoxifen inhibits the invasion capacity of mesenchymal-like MCs involves the inhibition of matrix metalloproteinase (MMP)-2 [155]. Furthermore, blocking of MMP-2 in MCs has been shown to reduce invasion by gastric cancer cells *in vitro* [156]. On this note, adhesion of ovarian cancer cells to the mesothelium is mediated by MMPs [85,101] and their inhibition in ovarian cancer cells prior to inoculation in mice resulted in reduced metastasis. Therefore, strategies directed towards MMP inhibition would also be of interest in the treatment of peritoneal metastases and may prevent the accumulation of MC-derived CAFs in the submesothelial stroma [157,158,159].

Recently, it has been demonstrated that the invasion capacity of MCs that have undergone a MMT is governed, at least partially, by the VEGF/VEGF receptors/co-receptors axis. It was shown that blocking antibodies directed against VEGF or the co-receptor neuropilin-1 efficiently reduced the invasion of MCs *in vitro* [128]. With regard to VEGF, its enhanced expression has been identified as one of the most evident effects associated to MMT during peritoneal metastasis [9]. Therefore, therapeutic intervention may also be directed at preventing peritoneal tumor vascularization via interrupting VEGF expression or its effects on the stromal endothelial cell [160]. Various anti-angiogenic therapies directed against VEGF or its receptors are presently being considered for the treatment of advanced abdominal cancers [161,162]. The use of Bevacizumab, a monoclonal antibody targeting VEGF, showed an improvement of patient survival in both colorectal [163] and ovarian cancer [164].

It is important to note that reports of MMT in peritoneal carcinomatosis are very recent. Specific treatments still require development; however, therapeutic options for interfering with the MMT in peritoneal dialysis could be considered for the metastasis scenario, since the effects of MMT (MC invasion, fibroblast accumulation, ECM deposition and angiogenesis) in the peritoneum seem to be similar in both pathologies.

## References

[1] De Cuba E.M., Kwakman R., van Egmond M., Bosch L.J., Bonjer H.J., Meijer G.A., te Velde E.A. (2012). Understanding molecular mechanisms in peritoneal dissemination of colorectal cancer: Future possibilities for personalised treatment by use of biomarkers. Virchows Arch..

[2] Tan D.S., Agarwal R., Kaye S.B. (2006). Mechanisms of transcoelomic metastasis in ovarian cancer. Lancet Oncol..

[3] Sohaib S.A., Houghton S.L., Meroni R., Rockall A.G., Blake P., Reznek R.H. (2007). Recurrent endometrial cancer: Patterns of recurrent disease and assessment of prognosis. Clin. Radiol..

[4] Koppe M.J., Boerman O.C., Oyen W.J., Bleichrodt R.P. (2006). Peritoneal carcinomatosis of colorectal origin: Incidence and current treatment strategies. Ann. Surg..

[5] Montori G., Coccolini F., Ceresoli M., Catena F., Colaianni N., Poletti E., Ansaloni L. (2014). The treatment of peritoneal carcinomatosis in advanced gastric cancer: State of the art. Int. J. Surg. Oncol..

[6] Glehen O., Cotte E., Brigand C., Arvieux C., Sayag-Beaujard A.C., Gilly F.N. (2006). Nouveautés thérapeutiques dans la prise en charge des carcinoses péritonéales d'origine digestive: Chirurgie de cytoréduction et chimiothérapie intrapéritonéale. [Therapeutic innovations in the management of peritoneal carcinomatosis from digestive origin: Cytoreductive surgery and intraperitoneal chemotherapy]. Rev. Med. Interne.

[7] Bakrin N., Classe J.M., Pomel C., Gouy S., Chene G., Glehen O. (2014). Hyperthermic intraperitoneal chemotherapy (HIPEC) in ovarian cancer. J. Visc. Surg..

[8] Glockzin G., Schlitt H.J., Piso P. (2009). Peritoneal carcinomatosis: Patients selection, perioperative complications and quality of life related to cytoreductive surgery and hyperthermic intraperitoneal chemotherapy. World J. Surg. Oncol..

[9] Sandoval P., Jiménez-Heffernan J.A., Rynne-Vidal A., Pérez-Lozano M.L., Gilsanz A., Ruiz-Carpio V., Reyes R., García-Bordas J., Stamatakis K., Dotor J. (2013). Carcinoma-associated fibroblasts derive from mesothelial cells via mesothelial to mesenchymal transition in peritoneal metastasis. J. Pathol..

[10] Chambers A.F., Groom A.C., MacDonald I.C. (2002). Dissemination and growth of cancer cells in metastatic sites. Nat. Rev. Cancer.

[11] Mendoza M., Khanna C. (2009). Revisiting the seed and soil in cancer metastasis. Int. J. Biochem. Cell Biol..

[12] Mathot L., Stenninger J. (2012). Behavior of seeds and soil in the mechanism of metastasis: A deeper understanding. Cancer Sci..

[13] Di Paolo N., Sacchi G. (2000). Atlas of peritoneal histology. Perit. Dial. Int..

[14] Hanahan D., Weinberg R.A. (2011). Hallmarks of cancer: The next generation. Cell.

[15] Bhome R., Bullock M.D., Al Saihati H.A., Goh R.W., Primrose J.N., Sayan A.E., Mirnezami A.H. (2015). A top-down view of the tumor microenvironment: Structure, cells and signaling. Front. Cell Dev. Biol..

[16] Kojima M., Higuchi Y., Yokota M., Ishii G., Saito N., Aoyagi K., Sasaki H., Ochiai A. (2014). Human subperitoneal fibroblast and cancer cell interaction creates microenvironment that enhances tumor progression and metastasis. PLoS ONE.

[17] Jimenez-Heffernan J.A., Aguilera A., Aroeira L.S., Lara-Pezzi E., Bajo M.A., del Peso G., Ramirez M., Gamallo C., Sanchez-Tomero J.A., Alvarez V. (2004). Immunohistochemical characterization of fibroblast subpopulations in normal peritoneal tissue and in peritoneal dialysis-induced fibrosis. Virchows Arch..

[18] Chegini N. (2008). TGF-beta system: The principal profibrotic mediator of peritoneal adhesion formation. Semin. Reprod. Med..

[19] Orimo A., Weinberg R.A. (2006). Stromal fibroblasts in cancer: A novel tumor-promoting cell type. Cell Cycle.

[20] Desmoulière A., Gabbiani G. (1994). Modulation of fibroblastic cytoskeletal features during pathological situations: The role of extracellular matrix and cytokines. Cell Motil. Cytoskeleton.

[21] Desmoulière A., Geinoz A., Gabbiani F., Gabbiani G. (1993). Transforming growth factor-beta1 induces a-smoothmuscle actin expression in granulation tissue myofibroblasts and in quiescent and growing cultured fibroblasts. J. Cell Biol..

[22] Lazard D., Sastre X., Frid M.G., Glukhova M.A., Thiery J.-P., Koteliansky V.E. (1993). Expression of smooth muscle-specific proteins in myoepithelium and stromal myofibroblasts ofnormal and malignant human breast tissue. Proc. Natl. Acad. Sci. USA.

[23] Calon A., Espinet E., Palomo-Ponce S., Tauriello D.V.F., Iglesias M., Céspedes M.V., Sevillano M., Nadal C., Jung P., Zhang X.H.F. (2012). Dependency of Colorectal Cancer on a TGF-β-Driven Program in Stromal Cells for Metastasis Initiation. Cancer Cell.

[24] Wikberg M.L., Edin S., Lundberg I.V., van Guelpen B., Dahlin A.M., Rutegard J., Stenling R., Oberg A., Palmqvist R. (2013). High intratumoral expression of fibroblast activation protein (FAP) in colon cancer is associated with poorer patient prognosis. Tumor Biol..

[25] Strutz F., Okada H., Lo C.W., Danoff T., Carone R.L., Tomaszewski J.E., Neilson E.G. (1995). Identification and characterization of a fibroblast marker: FSP1. J. Cell Biol..

[26] Polanska U.M., Orimo A. (2013). Carcinoma-associated fibroblasts: Non-neoplastic tumor-promoting mesenchymal cells. J. Cell. Physiol..

[27] Wels J., Kaplan R.N., Rafii S., Lyden D. (2008). Migratory neighbors and distant invaders: Tumor-associated niche cells. Genes Dev..

[28] Bhowmick N.A., Neilson E.G., Moses H.L. (2004). Stromal fibroblasts in cancer initiation and progression. Nature.

[29] Cirri P., Chiarugi P. (2012). Cancer-associated-fibroblasts and tumor cells: A diabolic liaison driving cancer progression. Cancer Metastasis Rev..

[30] Desmoulière A., Guyot C., Gabbiani G. (2004). The stroma reaction myofibroblast: A key player in the control of tumor cell behavior. Int. J. Dev. Biol..

[31] Kalluri R., Zeisberg M. (2006). Fibroblasts in cancer. Nat. Rev. Cancer.

[32] Lopez-Novoa J.M., Nieto M.A. (2009). Inflammation and EMT: An alliance towards organ fibrosis and cancer progression. EMBO Mol. Med..

[33] Potenta S., Zeisberg E., Kalluri R. (2008). The role of endothelial-to-mesenchymal transition in cancer progression. Br. J. Cancer.

[34] Zeisberg E.M., Potenta S.E., Sugimoto H., Zeisberg M., Kalluri R. (2008). Fibroblasts in kidney fibrosis emerge via endothelial-to-mesenchymal transition. J. Am. Soc. Nephrol..

[35] Yanez-Mo M., Lara-Pezzi E., Selgas R., Ramirez-Huesca M., Dominguez-Jimenez C., Jimenez-Heffernan J.A., Aguilera A., Sanchez-Tomero J.A., Bajo M.A., Alvarez V. (2003). Peritoneal dialysis and epithelial-to-mesenchymal transition of mesothelial cells. N. Engl. J. Med..

[36] Karki S., Surolia R., Hock T.D., Guroji P., Zolak J.S., Duggal R., Ye T., Thannickal V.J., Antony V.B. (2014). Wilms’ tumor 1 (Wt1) regulates pleural mesothelial cell plasticity and transition into myofibroblasts in idiopathic pulmonary fibrosis. FASEB J..

[37] Li Y., Wang J., Asahina K. (2013). Mesothelial cells give rise to hepatic stellate cells and myofibroblasts via mesothelial-mesenchymal transition in liver injury. Proc. Natl. Acad. Sci. USA.

[38] Ruiz-Villalba A., Simón A.M., Pogontke C., Castillo M.I., Abizanda G., Pelacho B., Sánchez-Domínguez R., Segovia J.C., Prósper F., Pérez-Pomares J.M. (2015). Interacting resident epicardium-derived fibroblasts and recruited bone marrow cells form myocardial infarction scar. J. Am. Coll. Cardiol..

[39] Deb A., Ubil E. (2014). Cardiac fibroblast in development and wound healing. J. Mol. Cell. Cardiol..

[40] Miao Z.-F., Zhao T.-T., Wang Z.-N., Miao F., Xu Y.-Y., Mao X.-Y., Gao J., Xu H.-M. (2014). Tumor-associated mesothelial cells are negative prognostic factors in gastric cancer and promote peritoneal dissemination of adherent gastric cancer cells by chemotaxis. Tumor Biol..

[41] López-Cabrera M. (2014). Mesenchymal conversion of mesothelial cells is a key event in the pathophysiology of the peritoneum during peritoneal dialysis. Adv. Med..

[42] Kalluri R., Weinberg R.A. (2009). The basics of epithelial-mesenchymal transition. J. Clin. Investig..

[43] Devuyst O., Margetts P.J., Topley N. (2010). The pathophysiology of the peritoneal membrane. J. Am. Soc. Nephrol..

[44] Margetts P.J., Bonniaud P. (2003). Basic mechanisms and clinical implications of peritoneal fibrosis. Perit. Dial. Int..

[45] Aroeira L.S., Aguilera A., Sanchez-Tomero J.A., Bajo M.A., del Peso G., Jimenez-Heffernan J.A., Selgas R., Lopez Cabrera M. (2007). Epithelial to mesenchymal transition and peritoneal membrane failure in peritoneal dialysis patients: Pathologic significance and potential therapeutic interventions. J. Am. Soc. Nephrol..

[46] Aguilera A., Yanez-Mo M., Selgas R., Sanchez-Madrid F., Lopez Cabrera M. (2005). Epithelial to mesenchymal transition as a triggering factor of peritoneal membrane fibrosis and angiogenesis in peritoneal dialysis patients. Curr. Opin. Investig. Drugs.

[47] Ayantunde A.A., Parsons S.L. (2007). Pattern and prognostic factors in patients with malignant ascites: A retrospective study. Ann. Oncol..

[48] Parsons S.L., Lang M.W., Steele R.J. (1996). Malignant ascites: A 2-year review from a teaching hospital. Eur. J. Surg. Oncol..

[49] Ayhan A., Gultekin M., Taskiran C., Dursun P., Firat P., Bozdag G., Celik N.Y., Yuce K. (2007). Ascites and epithelial ovarian cancers: A reappraisal with respect to different aspects. Int. J. Gynecol. Cancer.

[50] Sheid B. (1992). Angiogenic effects of macrophages isolated from ascitic fluid aspirated from women with advanced ovarian cancer. Cancer Lett..

[51] Milliken D., Scotton C., Raju S., Balkwill F., Wilson J. (2002). Analysis of chemokines and chemokine receptor expression in ovarian cancer ascites. Clin. Cancer Res..

[52] Choi D.-S., Park J.O., Jang S.C., Yoon Y.J., Jung J.W., Choi D.-Y., Kim J.-W., Kang J.S., Park J., Hwang D. (2011). Proteomic analysis of microvesicles derived from human colorectal cancer ascites. Proteomics.

[53] Graves L.E., Ariztia E.V., Navari J.R., Matzel H.J., Stack M.S., Fishman D.A. (2004). Proinvasive properties of ovarian cancer ascites-derived membrane vesicles. Cancer Res..

[54] Matte I., Lane D., Laplante C., Rancourt C., Piché A. (2012). Profiling of cytokines in human epithelial ovarian cancer ascites. Am. J. Cancer Res..

[55] Matte I., Lane D., Laplante C., Garde-Granger P., Rancourt C., Piché A. (2014). Ovarian cancer ascites enhance the migration of patient-derived peritoneal mesothelial cells viacMet pathway through HGF-dependent and -independent mechanisms. Int. J. Cancer.

[56] Sowter H.M., Corps A.N., Smith S.K. (1999). Hepatocyte growth factor (HGF) in ovarian epithelial tumor fluids stimulates the migration of ovarian carcinoma cells. Int. J. Cancer.

[57] Nash M.A., Lenzi R., Edwards C.L., Kavanagh J.J., Kudelka A.P., Verschraegen C.F., Platsoucas C.D., Freedman R.S. (1998). Differential expression of cytokine transcripts in human epithelial ovarian carcinoma by solid tumor specimens, peritoneal exudate cells containing tumor, tumor-infiltrating lymphocyte (TIL)-derived T cell lines and established tumor cell lines. Clin. Exp. Immunol..

[58] Stadlmann S., Amberger A., Pollheimer J., Gastl G., Offner F.A., Margreiter R., Zeimet A.G. (2005). Ovarian carcinoma cells and IL-1beta-activated human peritoneal mesothelial cells are possible sources of vascular endothelial growth factor in inflammatory and malignant peritoneal effusions. Gynecol. Oncol..

[59] Manenti L., Paganoni P., Floriani I., Landoni F., Torri V., Buda A., Taraboletti G., Labianca R., Belotti D., Giavazzi R. (2003). Expression levels of vascular endothelial growth factor, matrix metalloproteinases 2 and 9 and tissue inhibitor of metalloproteinases 1 and 2 in the plasma of patients with ovarian carcinoma. Eur. J. Cancer.

[60] Santin A.D., Bellone S., Ravaggi A., Roman J., Smith C.V., Pecorelli S., Cannon M.J., Parham G.P. (2001). Increased levels of interleukin-10 and transforming growth factor-beta in the plasma and ascitic fluid of patients with advanced ovarian cancer. BJOG.

[61] Moradi M.M., Carson L.F., Twiggs L.B., Weinberg J.B., Haney A.F., Ramakrishnan S. (1993). Serum and ascitic fluid levels of interleukin-1, interleukin-6, and tumor necrosis factor-alpha in patients with ovarian epithelial cancer. Cancer.

[62] Giuntoli R.L., Webb T.J., Zoso A., Rogers O., Diaz-Montes T.P., Bristow R.E., Oelke M. (2009). Ovarian cancer-associated ascites demonstrates altered immune environment: Implications for antitumor immunity. Anticancer Res..

[63] Masunaga Y., Muto S., Asakura S., Akimoto T., Homma S., Kusano E., Asano Y. (2003). Ascites from patients with encapsulating peritoneal sclerosis augments NIH/3T3 fibroblast proliferation. Ther. Apher. Dial..

[64] Fredj S., Bescond J., Louault C., Delwail A., Lecron J.-C., Potreau D. (2005). Role of interleukin-6 in cardiomyocyte/cardiac fibroblast interactions during myocyte hypertrophy and fibroblast proliferation. J. Cell. Physiol..

[65] Demir A.Y., Groothuis P.G., Dunselman G.A.J., Schurgers L., Evers J.L.H., de Goeij A.F.P.M. (2005). Molecular characterization of soluble factors from human menstrual effluent that induce epithelial to mesenchymal transitions in mesothelial cells. Cell Tissue Res..

[66] Freedman R.S., Deavers M., Liu J., Wang E. (2004). Peritoneal inflammation—A microenvironment for Epithelial Ovarian Cancer (EOC). J. Transl. Med..

[67] Kolomeyevskaya N., Eng K.H., Khan A.N.H., Grzankowski K.S., Singel K.L., Moysich K., Segal B.H. (2015). Cytokine profiling of ascites at primary surgery identifies an interaction of tumor necrosis factor-α and interleukin-6 in predicting reduced progression-free survival in epithelial ovarian cancer. Gynecol. Oncol..

[68] Matte I., Lane D., Bachvarov D., Rancourt C., Piche A. (2014). Role of malignant ascites on human mesothelial cells and their gene expression profiles. BMC Cancer.

[69] Lane D., Matte I., Rancourt C., Piché A. (2011). Prognostic significance of IL-6 and IL-8 ascites levels in ovarian cancer patients. BMC Cancer.

[70] Ono Y.J., Hayashi M., Tanabe A., Hayashi A., Kanemura M., Terai Y., Ohmichi M. (2015). Estradiol-mediated hepatocyte growth factor is involved in the implantation of endometriotic cells via the mesothelial-to-mesenchymal transition in the peritoneum. Am. J. Physiol. Endocrinol. Metab..

[71] Nakamura M., Ono Y.J., Kanemura M., Tanaka T., Hayashi M., Terai Y., Ohmichi M. (2015). Hepatocyte growth factor secreted by ovarian cancer cells stimulates peritoneal implantation via the mesothelial-mesenchymal transition of the peritoneum. Gynecol. Oncol..

[72] Thibault B., Castells M., Delord J.P., Couderc B. (2014). Ovarian cancer microenvironment: Implications for cancer dissemination and chemoresistance acquisition. Cancer Metastasis Rev..

[73] Elliott R.L. (2005). Role of transforming growth factor beta in human cancer. J. Clin. Oncol..

[74] Loureiro J., Aguilera A., Selgas R., Sandoval P., Albar-Vizcaino P., Pérez-Lozano M.L., Ruiz-Carpio V., Majano P.L., Lamas S., Rodriguez-Pascual F. (2011). Blocking TGF-1 protects the peritoneal membrane from dialysate-induced damage. J. Am. Soc. Nephrol..

[75] Yamamura S., Matsumura N., Mandai M., Huang Z., Oura T., Baba T., Hamanishi J., Yamaguchi K., Kang H.S., Okamoto T. (2011). The activated transforming growth factor-beta signaling pathway in peritoneal metastases is a potential therapeutic target in ovarian cancer. Int. J. Cancer.

[76] Miao Z.-F., Zhao T.-T., Wang Z.-N., Miao F., Xu Y.-Y., Mao X.-Y., Gao J., Wu J.-H., Liu X.-Y., You Y. (2013). Transforming growth factor-beta1 signaling blockade attenuates gastric cancer cell-induced peritoneal mesothelial cell fibrosis and alleviates peritoneal dissemination both *in vitro* and *in vivo*. Tumor Biol..

[77] Théry C., Zitvogel L., Amigorena S. (2002). Exosomes: Composition, biogenesis and function. Nat. Rev. Immunol..

[78] Peng P., Yan Y., Keng S. (2011). Exosomes in the ascites of ovarian cancer patients: Origin and effects on anti-tumor immunity. Oncol. Rep..

[79] Cappellesso R., Tinazzi A., Giurici T., Simonato F., Guzzardo V., Ventura L., Crescenzi M., Chiarelli S., Fassina A. (2014). Programmed cell death 4 and microRNA 21 inverse expression is maintained in cells and exosomes from ovarian serous carcinoma effusions. Cancer Cytopathol..

[80] Tokuhisa M., Ichikawa Y., Kosaka N., Ochiya T., Yashiro M., Hirakawa K., Kosaka T., Makino H., Akiyama H., Kunisaki C. (2015). Exosomal miRNAs from peritoneum lavage fluid as potential prognostic biomarkers of peritoneal metastasis in gastric cancer. PLoS ONE.

[81] Vaksman O., Trope C., Davidson B., Reich R. (2014). Exosome-derived miRNAs and ovarian carcinoma progression. Carcinogenesis.

[82] Selcuklu S.D., Donoghue M.T.A., Spillane C. (2009). miR-21 as a key regulator of oncogenic processes. Biochem. Soc. Trans..

[83] Na D., Lv Z.D., Liu F.N., Xu Y., Jiang C.G., Sun Z., Miao Z.F., Li F., Xu H.M. (2010). Transforming growth factor beta1 produced in autocrine/paracrine manner affects the morphology and function of mesothelial cells and promotes peritoneal carcinomatosis. Int. J. Mol. Med..

[84] Heath R.M., Jayne D.G., O’Leary R., Morrison E.E., Guillou P.J. (2004). Tumor-induced apoptosis in human mesothelial cells: A mechanism of peritoneal invasion by Fas Ligand/Fas interaction. Br. J. Cancer.

[85] Burleson K.M., Hansen L.K., Skubitz A.P. (2004). Ovarian carcinoma spheroids disaggregate on type I collagen and invade live human mesothelial cell monolayers. Clin. Exp. Metastasis.

[86] Iwanicki M.P., Davidowitz R.A., Ng M.R., Besser A., Muranen T., Merritt M., Danuser G., Ince T., Brugge J.S. (2011). Ovarian cancer spheroids use myosin-generated force to clear the mesothelium. Cancer Discov..

[87] Davidowitz R.A., Selfors L.M., Iwanicki M.P., Elias K.M., Karst A., Piao H., Ince T.A., Drage M.G., Dering J., Konecny G.E. (2014). Mesenchymal gene program-expressing ovarian cancer spheroids exhibit enhanced mesothelial clearance. J. Clin. Investig..

[88] Clark R., Krishnan V., Schoof M., Rodriguez I., Theriault B., Chekmareva M., Rinker-Schaeffer C. (2013). Milky spots promote ovarian cancer metastatic colonization of peritoneal adipose in experimental models. Am. J. Pathol..

[89] Sodek K.L., Murphy K.J., Brown T.J., Ringuette M.J. (2012). Cell-cell and cell-matrix dynamics in intraperitoneal cancer metastasis. Cancer Metastasis Rev..

[90] De Vlieghere E., Gremonprez F., Verset L., Marien L., Jones C.J., de Craene B., Berx G., Descamps B., Vanhove C., Remon J.P. (2015). Tumor-environment biomimetics delay peritoneal metastasis formation by deceiving and redirecting disseminated cancer cells. Biomaterials.

[91] Ziprin P., Ridgway P.F., Pfistermuller K.L., Peck D.H., Darzi A.W. (2003). ICAM-1 mediated tumor-mesothelial cell adhesion is modulated by IL-6 and TNF-alpha: A potential mechanism by which surgical trauma increases peritoneal metastases. Cell Commun. Adhes..

[92] Rump A., Morikawa Y., Tanaka M., Minami S., Umesaki N., Takeuchi M., Miyajima A. (2004). Binding of ovarian cancer antigen CA125/MUC16 to mesothelin mediates cell adhesion. J. Biol. Chem..

[93] Alkhamesi N.A., Ziprin P., Pfistermuller K., Peck D.H., Darzi A.W. (2005). ICAM-1 mediated peritoneal carcinomatosis, a target for therapeutic intervention. Clin. Exp. Metastasis.

[94] Casey R.C., Skubitz A.P. (2000). CD44 and beta1 integrins mediate ovarian carcinoma cell migration toward extracellular matrix proteins. Clin. Exp. Metastasis.

[95] Ksiazek K., Mikula-Pietrasik J., Catar R., Dworacki G., Winckiewicz M., Frydrychowicz M., Dragun D., Staniszewski R., Jorres A., Witowski J. (2010). Oxidative stress-dependent increase in ICAM-1 expression promotes adhesion of colorectal and pancreatic cancers to the senescent peritoneal mesothelium. Int. J. Cancer.

[96] Yu G., Tang B., Yu P.W., Peng Z.H., Qian F., Sun G. (2010). Systemic and peritoneal inflammatory response after laparoscopic-assisted gastrectomy and the effect of inflammatory cytokines on adhesion of gastric cancer cells to peritoneal mesothelial cells. Surg. Endosc..

[97] Wagner B.J., Lob S., Lindau D., Horzer H., Guckel B., Klein G., Glatzle J., Rammensee H.G., Brucher B.L., Konigsrainer A. (2011). Simvastatin reduces tumor cell adhesion to human peritoneal mesothelial cells by decreased expression of VCAM-1 and beta1 integrin. Int. J. Oncol..

[98] Chen C.N., Chang C.C., Lai H.S., Jeng Y.M., Chen C.I., Chang K.J., Lee P.H., Lee H. (2015). Connective tissue growth factor inhibits gastric cancer peritoneal metastasis by blocking integrin alpha3beta1-dependent adhesion. Gastric Cancer.

[99] Jiang C.-G., Lv L., Liu F.-R., Wang Z.-N., Na D., Li F., Li J.-B., Sun Z., Xu H.-M. (2013). Connective tissue growth factor is a positive regulator of epithelial-mesenchymal transition and promotes the adhesion with gastric cancer cells in human peritoneal mesothelial cells. Cytokine.

[100] Kenny H.A., Lengyel E. (2009). MMP-2 functions as an early response protein in ovarian cancer metastasis. Cell Cycle.

[101] Kenny H.A., Kaur S., Coussens L.M., Lengyel E. (2008). The initial steps of ovarian cancer cell metastasis are mediated by MMP-2 cleavage of vitronectin and fibronectin. J. Clin. Investig..

[102] Ranieri D., Raffa S., Parente A., Rossi Del Monte S., Ziparo V., Torrisi M.R. (2013). High adhesion of tumor cells to mesothelial monolayers derived from peritoneal wash of disseminated gastrointestinal cancers. PLoS ONE.

[103] Slack-Davis J.K., Atkins K.A., Harrer C., Hershey E.D., Conaway M. (2009). Vascular cell adhesion molecule-1 is a regulator of ovarian cancer peritoneal metastasis. Cancer Res..

[104] Kenny H.A., Chiang C.-Y., White E.A., Schryver E.M., Habis M., Romero I.L., Ladanyi A., Penicka C.V., George J., Matlin K. (2014). Mesothelial cells promote early ovarian cancer metastasis through fibronectin secretion. J. Clin. Investig..

[105] Strippoli R., Benedicto I., Pérez-Lozano M.L., Pellinen T., Sandoval P., López-Cabrera M., del Pozo M.A. (2012). Inhibition of transforming growth factor-activated kinase 1 (TAK1) blocks and reverses epithelial to mesenchymal transition of mesothelial cells. PLoS ONE.

[106] Satoyoshi R., Kuriyama S., Aiba N., Yashiro M., Tanaka M. (2015). Asporin activates coordinated invasion of scirrhous gastric cancer and cancer-associated fibroblasts. Oncogene.

[107] Satoyoshi R., Aiba N., Yanagihara K., Yashiro M., Tanaka M. (2015). Tks5 activation in mesothelial cells creates invasion front of peritoneal carcinomatosis. Oncogene.

[108] Kenny H.A., Krausz T., Yamada S.D., Lengyel E. (2007). Use of a novel 3D culture model to elucidate the role of mesothelial cells, fibroblasts and extra-cellular matrices on adhesion and invasion of ovarian cancer cells to the omentum. Int. J. Cancer.

[109] Cai J., Tang H., Xu L., Wang X., Yang C., Ruan S., Guo J., Hu S., Wang Z. (2011). Fibroblasts in omentum activated by tumor cells promote ovarian cancer growth, adhesion and invasiveness. Carcinogenesis.

[110] Mikula-Pietrasik J., Sosinska P., Kucinska M., Murias M., Maksin K., Malinska A., Ziolkowska A., Piotrowska H., Wozniak A., Ksiazek K. (2014). Peritoneal mesothelium promotes the progression of ovarian cancer cells *in vitro* and in a mice xenograft model *in vivo*. Cancer Lett..

[111] Tsukada T., Fushida S., Harada S., Yagi Y., Kinoshita J., Oyama K., Tajima H., Fujita H., Ninomiya I., Fujimura T. (2012). The role of human peritoneal mesothelial cells in the fibrosis and progression of gastric cancer. Int. J. Oncol..

[112] Schropfer A., Kammerer U., Kapp M., Dietl J., Feix S., Anacker J. (2010). Expression pattern of matrix metalloproteinases in human gynecological cancer cell lines. BMC Cancer.

[113] John A., Tuszynski G. (2001). The role of matrix metalloproteinases in tumor angiogenesis and tumor metastasis. Pathol. Oncol. Res..

[114] Kessenbrock K., Plaks V., Werb Z. (2010). Matrix metalloproteinases: Regulators of the tumor microenvironment. Cell.

[115] Al-Alem L., Curry T.E.J. (2015). Ovarian cancer: Involvement of the matrix metalloproteinases. Reproduction.

[116] Paulsson J., Micke P. (2014). Prognostic relevance of cancer-associated fibroblasts in human cancer. Semin. Cancer Biol..

[117] Taddei M.L., Giannoni E., Comito G., Chiarugi P. (2013). Microenvironment and tumor cell plasticity: An easy way out. Cancer Lett..

[118] Margetts P.J., Bonniaud P., Liu L., Hoff C.M., Holmes C.J., West-Mays J.A., Kelly M.M. (2005). Transient overexpression of TGF-b1 induces epithelial mesenchymal transition in the rodent peritoneum. J. Am. Soc. Nephrol..

[119] Zhu Z., Yao J., Wang F., Xu Q. (2002). TNF-alpha and the phenotypic transformation of human peritoneal mesothelial cell. Chin. Med. J..

[120] Yu Q., Stamenkovic I. (2000). Cell surface-localized matrix metalloproteinase-9 proteolytically activates TGF-beta and promotes tumor invasion and angiogenesis. Genes Dev..

[121] Belotti D., Calcagno C., Garofalo A., Caronia D., Riccardi E., Giavazzi R., Taraboletti G. (2008). Vascular endothelial growth factor stimulates organ-specific host matrix metalloproteinase-9 expression and ovarian cancer invasion. Mol. Cancer Res..

[122] Birkedal-Hansen H., Moore W.G., Bodden M.K., Windsor L.J., Birkedal-Hansen B., DeCarlo A., Engler J.A. (1993). Matrix metalloproteinases: A review. Crit. Rev. Oral Biol. Med..

[123] Lee I.K., Vansaun M.N., Shim J.H., Matrisian L.M., Gorden D.L. (2013). Increased metastases are associated with inflammation and matrix metalloproteinase-9 activity at incision sites in a murine model of peritoneal dissemination of colorectal cancer. J. Surg. Res..

[124] Lee M.A., Park J.H., Rhyu S.Y., Oh S.T., Kang W.K., Kim H.N. (2014). Wnt3a expression is associated with MMP-9 expression in primary tumor and metastatic site in recurrent or stage IV colorectal cancer. BMC Cancer.

[125] Liotta L.A., Kohn E.C. (2001). The microenvironment of the tumor-host interface. Nature.

[126] Kitadai Y. (2010). Cancer-stromal cell interaction and tumor angiogenesis in gastric cancer. Cancer Microenviron..

[127] Winiarski B.K., Cope N., Alexander M., Pilling L.C., Warren S., Acheson N., Gutowski N.J., Whatmore J.L. (2014). Clinical relevance of increased endothelial and mesothelial expression of proangiogenic proteases and VEGFA in the omentum of patients with metastatic ovarian high-grade serous carcinoma. Transl. Oncol..

[128] Pérez-Lozano M.L., Sandoval P., Rynne-Vidal A., Aguilera A., Jiménez-Heffernan J.A., Albar-Vizcaíno P., Majano P.L., Sánchez-Tomero J.A., Selgas R., López-Cabrera M. (2013). Functional relevance of the switch of VEGF receptors/co-receptors during peritoneal dialysis-induced mesothelial to mesenchymal transition. PLoS ONE.

[129] Aroeira L.S., Aguilera A., Selgas R., Ramirez-Huesca M., Perez Lozano M.L., Cirugeda A., Bajo M.A., del Peso G., Sanchez-Tomero J.A., Jimenez-Heffernan J.A. (2005). Mesenchymal conversion of mesothelial cells as a mechanism responsible for high solute transport rate in peritoneal dialysis: Role of vascular endothelial growth factor. Am. J. Kidney Dis..

[130] Sako A., Kitayama J., Yamaguchi H., Kaisaki S., Suzuki H., Fukatsu K., Fujii S., Nagawa H. (2003). Vascular endothelial growth factor synthesis by human omental mesothelial cells is augmented by fibroblast growth factor-2: Possible role of mesothelial cell on the development of peritoneal metastasis. J. Surg. Res..

[131] Sako A., Kitayama J., Koyama H., Ueno H., Uchida H., Hamada H., Nagawa H. (2004). Transduction of soluble Flt-1 gene to peritoneal mesothelial cells can effectively suppress peritoneal metastasis of gastric cancer. Cancer Res..

[132] Micke P., Ostman A. (2005). Exploring the tumor environment: Cancer-associated fibroblasts as targets in cancer therapy. Expert Opin. Ther. Targets.

[133] Yoshimura A., Wakabayashi Y., Mori T. (2010). Cellular and molecular basis for the regulation of inflammation by TGF-beta. J. Biochem..

[134] Wang X., Nie J., Jia Z., Feng M., Zheng Z., Chen W., Li X., Peng W., Zhang S., Sun L. (2010). Impaired TGF-beta signalling enhances peritoneal inflammation induced by *E. coli* in rats. Nephrol. Dial. Transplant..

[135] Loureiro J., Schilte M., Aguilera A., Albar-Vizcaino P., Ramirez-Huesca M., Perez Lozano M.L., Gonzalez-Mateo G., Aroeira L.S., Selgas R., Mendoza L. (2010). BMP-7 blocks mesenchymal conversion of mesothelial cells and prevents peritoneal damage induced by dialysis fluid exposure. Nephrol. Dial. Transpl..

[136] Vargha R., Endemann M., Kratochwill K., Riesenhuber A., Wick N., Krachler A.-M., Malaga-Dieguez L., Aufricht C. (2006). *Ex vivo* reversal of *in vivo* transdifferentiation in mesothelial cells grown from peritoneal dialysate effluents. Nephrol. Dial. Transplant..

[137] Strippoli R., Benedicto I., Lozano M.L.P., Cerezo A., López-Cabrera M., del Pozo M.A. (2008). Epithelial-to-mesenchymal transition of peritoneal mesothelial cells is regulated by an ERK/NF-κB/Snail1 pathway. Dis. Model Mech..

[138] Strippoli R., Benedicto I., Foronda M., Pérez-Lozano M.L., Sánchez-Perales S., López-Cabrera M., del Pozo M.A. (2010). p38 maintains E-cadherin expression by modulating TAK1-NF-kappa B during epithelial-to-mesenchymal transition. J. Cell Sci..

[139] Jang Y.-H., Shin H.-S., Choi H.S., Ryu E.-S., Kim M.J., Min S.K., Lee J.-H., Lee H.K., Kim K.-H., Kang D.-H. (2012). Effects of dexamethasone on the TGF-b1-induced epithelial-to-mesenchymal transition in human peritoneal mesothelial cells. Lab. Investig..

[140] Aroeira L.S., Lara-Pezzi E., Loureiro J., Aguilera A., Ramírez-Huesca M., González-Mateo G., Pérez-Lozano M.L., Albar-Vizcaíno P., Bajo M.-A., del Peso G. (2009). Cyclooxygenase-2 mediates dialysate-induced alterations of the peritoneal membrane. J. Am. Soc. Nephrol..

[141] Fabbrini P., Schilte M.N., Zareie M., ter Wee P.M., Keuning E.D., Beelen R.H.J., van den Born J. (2009). Celecoxib treatment reduces peritoneal fibrosis and angiogenesis and prevents ultrafiltration failure in experimental peritoneal dialysis. Nephrol. Dial. Transpl..

[142] Menczer J. (2009). Cox-2 expression in ovarian malignancies: A review of the clinical aspects. Eur. J. Obstet. Gynecol. Reprod. Biol..

[143] Rizzo M.T. (2011). Cyclooxygenase-2 in oncogenesis. Clin. Chim. Acta.

[144] Arber N., Eagle C.J., Spicak J., Rácz I., Dite P., Hajer J., Zavoral M., Lechuga M.J., Gerletti P., Tang J. (2006). PreSAP Trial Investigators Celecoxib for the prevention of colorectal adenomatous polyps. N. Engl. J. Med..

[145] Baron J.A., Sandler R.S., Bresalier R.S., Quan H., Riddell R., Lanas A., Bolognese J.A., Oxenius B., Horgan K., Loftus S. (2006). APPROVe trial investigators a randomized trial of rofecoxib for the chemoprevention of colorectal adenomas. Gastroenterology.

[146] Midgley R.S., McConkey C.C., Johnstone E.C., Dunn J.A., Smith J.L., Grumett S.A., Julier P., Iveson C., Yanagisawa Y., Warren B. (2010). Phase III randomized trial assessing rofecoxib in the adjuvant setting of colorectal cancer: Final results of the VICTOR trial. J. Clin. Oncol..

[147] Solomon S.D., Pfeffer M.A., McMurray J.J.V., Fowler R., Finn P., Levin B., Eagle C., Hawk E., Lechuga M., Zauber A.G. (2006). APC and PreSAP Trial Investigators Effect of celecoxib on cardiovascular events and blood pressure in two trials for the prevention of colorectal adenomas. Circulation.

[148] Watanabe T., Hashimoto T., Sugino T., Soeda S., Nishiyama H., Morimura Y., Yamada H., Goodison S., Fujimori K. (2012). Production of IL1-beta by ovarian cancer cells induces mesothelial cell beta1-integrin expression facilitating peritoneal dissemination. J. Ovarian Res..

[149] Yokoyama Y., Sedgewick G., Ramakrishnan S. (2007). Endostatin binding to ovarian cancer cells inhibits peritoneal attachment and dissemination. Cancer Res..

[150] Mitra A.K., Sawada K., Tiwari P., Mui K., Gwin K., Lengyel E. (2011). Ligand-independent activation of c-Met by fibronectin and α(5)β(1)-integrin regulates ovarian cancer invasion and metastasis. Oncogene.

[151] Sawada K., Mitra A.K., Radjabi A.R., Bhaskar V., Kistner E.O., Tretiakova M., Jagadeeswaran S., Montag A., Becker A., Kenny H.A. (2008). Loss of E-cadherin promotes ovarian cancer metastasis via alpha 5-integrin, which is a therapeutic target. Cancer Res..

[152] Ksiazek K., Mikula-Pietrasik J., Korybalska K., Dworacki G., Jörres A., Witowski J. (2009). Senescent peritoneal mesothelial cells promote ovarian cancer cell adhesion. Am. J. Pathol..

[153] Ksiazek K. (2013). Mesothelial cell: A multifaceted model of aging. Ageing Res. Rev..

[154] Serkies K., Sinacki M., Jassem J. (2013). The role of hormonal factors and endocrine therapy in ovarian cancer. Contemp. Oncol. (Pozn).

[155] Loureiro J., Sandoval P., del Peso G., González-Mateo G., Fernández-Millara V., Santamaria B., Bajo M.A., Sánchez-Tomero J.A., Guerra-Azcona G., Selgas R. (2013). Tamoxifen ameliorates peritoneal membrane damage by blocking mesothelial to mesenchymal transition in peritoneal dialysis. PLoS ONE.

[156] Mizutani K., Kofuji K., Shirouzu K. (2000). The significance of MMP-1 and MMP-2 in peritoneal disseminated metastasis of gastric cancer. Surg. Today.

[157] Cathcart J., Pulkoski-Gross A., Cao J. (2015). Targeting matrix metalloproteinases in cancer: Bringing new life to old ideas. Genes Dis..

[158] Gialeli C., Theocharis A.D., Karamanos N.K. (2010). Roles of matrix metalloproteinases in cancer progression and their pharmacological targeting. FEBS J..

[159] Egeblad M., Werb Z. (2002). New functions for the matrix metalloproteinases in cancer progression. Nat. Rev. Cancer.

[160] Wu Q.J., Gong C.Y., Luo S.T., Zhang D.M., Zhang S., Shi H.S., Lu L., Yan H.X., He S.S., Li D.D. (2012). AAV-mediated human PEDF inhibits tumor growth and metastasis in murine colorectal peritoneal carcinomatosis model. BMC Cancer.

[161] Masoumi Moghaddam S., Amini A., Morris D.L., Pourgholami M.H. (2011). Significance of vascular endothelial growth factor in growth and peritoneal dissemination of ovarian cancer. Cancer Metastasis Rev..

[162] Gadducci A., Lanfredini N., Sergiampietri C. (2015). Antiangiogenic agents in gynecological cancer: State of art and perspectives of clinical research. Crit. Rev. Oncol. Hematol..

[163] Mulder K., Scarfe A., Chua N., Spratlin J. (2011). The role of bevacizumab in colorectal cancer: Understanding its benefits and limitations. Expert Opin. Biol. Ther..

[164] Perren T.J., Swart A.M., Pfisterer J., Ledermann J.A., Pujade-Lauraine E., Kristensen G., Carey M.S., Beale P., Cervantes A., Kurzeder C. (2011). A phase 3 trial of bevacizumab in ovarian cancer. N. Engl. J. Med..

